# A Convenient and Robust Test to Quantify Interocular Suppression for Children With Amblyopia

**DOI:** 10.1177/2041669519864971

**Published:** 2019-07-24

**Authors:** Hui Chen, Zhifen He, Jinling Xu, Yu Mao, Yunjie Liang, Danli Lin, Meiping Xu, Zhiyue Dai, Xiaoxin Chen, Jiawei Zhou, Xinping Yu

**Affiliations:** School of Ophthalmology and Optometry and Eye Hospital, Wenzhou Medical University, Wenzhou, Zhejiang, PR China

**Keywords:** interocular suppression, amblyopia, luminance

## Abstract

Interocular suppression was quantified by the interocular luminance difference that was needed when the two eyes were balanced in discriminating a black–white stripe formed butterfly stimulus, which was dichoptically presented through polarized glasses. Stronger interocular suppression was found in amblyopes than that in controls at both the near (33 cm, 0.95 ± 1.00 vs. 0.14 ± 0.18, *p* < .001) and far (5 m, 2.18 ± 0.97 vs. 0.24 ± 0.16, *p* < .001) viewing distances. The interocular suppression in amblyopes was significantly correlated with the interocular visual acuity difference, the visual acuity of amblyopic eye, the Worth-4-Dot test, and the stereo acuity at both the near and far distances (for all cases, *p* < .001). Our new test enables convenient and robust measurements of interocular suppression in children with amblyopia. The measured interocular suppression is in agreement with other clinical measures.

## Introduction

Amblyopia is a neurodevelopmental disorder of the visual system resulting from disruption to binocular visual experience during early childhood. It affects about 3% to 5% of the population and is one of the most common causes of monocular visual impairment (Attebo et al., 1998; He et al., 2004; Holmes & Clarke, 2006). The two eyes of amblyopes are imbalanced in binocular viewing, although the same levels of visual input enter both eyes. It is thought to be caused by a stronger suppression from the fellow-fixing eye to the amblyopic eye (Huang, Zhou, Lu, Feng, & Zhou, 2009; Huang, Zhou, Lu, & Zhou, 2011; Zhou et al., 2018). Several studies have shown that the depth of imbalanced interocular suppression is closely related to the degree of amblyopia and the loss of stereopsis (Baker, Meese, & Hess, 2008; Birch, 2013; Kwon et al., 2014; Kwon, Wiecek, Dakin, & Bex, 2015; Li et al., 2011; Zhou, Huang, & Hess, 2013). There is also evidence that antisuppression training (Hess, Mansouri, & Thompson, 2010b, 2011; Kelly et al., 2018) or optical treatment (Gao et al., 2018; Wang, Feng, Wang, Zhou, & Hess, 2018) benefits not only binocular but also monocular vision in amblyopia. Interocular suppression has been thought to play a primary role in amblyopia (Hess, Mansouri, & Thompson, 2010a), although this idea has been disputed (Bossi et al., 2017).

According to previous studies, the depth of imbalanced interocular suppression has been quantified with laboratory-based psychophysical procedures, including global coherent motion task (Mansouri, Thompson, & Hess, 2008), binocular phase combination task (Ding, Klein, & Levi, 2013; Huang et al., 2009; Kwon et al., 2015; Zhou, Huang, et al., 2013), dichoptic EDTRS task (Kwon et al., 2015), binocular rivalry task (Ooi & He, 2001), optokinetic nystagmus paradigm (Wen et al., 2018), dichoptic noise masking paradigm (Zhou et al., 2018), and so on (see a comparison of tests in Bossi, Hamm, Dahlmann-Noor, & Dakin, 2018). These techniques, although precise and quantitative, are not friendly for children in the clinical setting because they normally involve the use of complicated devices and/or take a long time to complete.

Several researchers have tried implementing the laboratory-based psychophysical procedures to measure interocular suppression in a child-friendly fashion. For example, Narasimhan, Harrison, and Giaschi (2012) added Disney characters from the movie *Finding Nemo* to make the global coherent motion task (Mansouri et al., 2008) child-friendly. This study placed the dichoptic global motion task within a fish-themed game with practice trials and staircase parameters altered for use with children of as young as 5 years old. Birch et al. (2016) have also shown that the dichoptic letter chart method is successful in testing the depth of imbalanced interocular suppression in children ranging from 3 to 12 years old. In these studies, the depth of imbalanced interocular suppression was quantified by the interocular contrast ratio where the two eyes were equally effective in binocular viewings. Recently, Zhou, Jia, Huang, and Hess (2013) showed that the depth of imbalanced interocular suppression in amblyopes could be modulated by introducing interocular luminance differences. For example, the two eyes of amblyope could be balanced in binocular combination if the luminance in the fellow eye was attenuated to some degree. This phenomenon can be accounted for by the luminance modulated contrast-gain control model (i.e., lower input luminance in the fellow eye reduced the contrast-gain of this eye, thus shifting the perceptual eye dominance toward the amblyopic eye). These results suggest that the depth of imbalanced interocular suppression can be quantified by the interocular luminance difference that is needed to balance the two eyes during binocular viewing.

Luminance-based suppression tests have actually been used in the clinical setting (e.g., the Sbisa bar (Crawford & Griffiths, 2015) and neutral density (ND) filter bars (Piano & Newsham, 2015). In these methods, patients are presented with increasing densities of ND filters or red-tinted absorbing filters (a *Sbisa bar*) over the nonamblyopic/dominant eye until there is a switch of ocular dominance to the amblyopic/nondominant eye. However, the test–retest reliability was an issue, even in adults (Piano & Newsham, 2015). These tests rely on the patient’s ability to detect and report a change in fixation through changes in light intensity or color, or to report diplopia, which can be difficult for patients with suppression. In this study, we designed a new version of luminance-based suppression test, in which a full contrast stimulus, rather than a light was used as the visual target. We show that this measure is convenient and robust for suppression measurement in clinic, and in agreement with other related clinical measures.

## Materials and Methods

### Participants

Twenty-nine children with a history of unilateral amblyopia (8.69 ± 2.42 years old; mean ± *SD*; A1–A29) and 20 age-matched normal controls (8.00 ± 1.89 years old) participated in the main study. We measured 20 of the children with a history of unilateral amblyopia (8.95 ± 2.37 years old; A1–A20) and 12 of the normal controls (8.50 ± 1.41 years old) twice in 1 to 2 weeks to assess the test–retest reliability. Another five amblyopes (16.8 ± 3.46 years old; A30–A34) participated in additional test; we assessed the relationship between the suppression index measured with our test and that measured with a classical binocular phase combination task (Ding & Sperling, 2006; Huang et al., 2009; Zhou, Huang, et al., 2013; Zhou, Jia, et al., 2013).

Clinical examinations included the best-corrected visual acuity, refractive errors, slit lamp examination, extraocular muscle movements, intraocular pressure, and ophthalmoscopic exam. Best-corrected visual acuity was tested using the Chinese Tumbling E Visual chart (Mou, 1966) at 5 m. Deviation was measured using a prism alternate cover test at distance of 5 m with corrected refractive errors. Cycloplegic refraction was measured with a table-mounted autorefractor (model KR-8900; Hasunuma-cho, Itabashi-ku, Tokyo, Japan).

Amblyopia was defined according to the Preferred Practice Protocol (PPP) of The American Academy of Ophthalmology (American Academy of Ophthalmology; Preferred Practice Patterns; Amblyopia PPP, September 2012; available at http://www.aao.org/ppp) and classified as one of the following: strabismic, anisometropic, or mixed (those that met the criteria for both types of amblyopia), with visual acuity in the amblyopic eye between 0.10 (logMAR) and 1.00 (logMAR), and 0.05 (logMAR) or better vision in the fellow eye. Exclusion criteria included: patients with organic eye disease, a history or evidence of cataract, glaucoma, retinal disorders, or laser treatment. The clinical details of patients are provided in Table 1. Treatment status was not considered for study enrollment. Some patients had been successfully treated and could have had an interocular visual acuity difference of less than 2 lines; details of the treatment history (e.g., refractive correction, occlusion) are provided in Table 1.

**Table 1.** Clinical Details of the Participants.

Obs	Age/Sex	Type	Refraction (OD/OS)	Visual Acuity (logMAR): OD/OS	Strabismus	Worth-4-Dot test^a^ far/near	Stereopsis (sec arc) far/near	History	
								Refractive correction	Occlusion/penalization
A1	5/M	Anis	+2.50/−3.50 × 180+1.75/−1.50 × 180	0.10/−0.10	Ø	0/0	200/60	Yes	No
A2	12/M	Anis	−0.25/−0.25 × 180+3.50/−0.50 × 180	0.00/0.15	Ø	2/0	800/800^b^	Yes	Yes
A3	6/M	Anis	+4.00/−0.75 × 180+0.75/−1.00 × 165	0.30/0.05	Ø	2/1	400/800	Yes	Yes
A4	9/F	Anis	+2.50/−0.50 × 180+5.00/−1.00 × 5	−0.10/0.10	Ø	0/0	100/60	Yes	Yes
A5	7/M	Anis	+7.25/−0.50 × 180+3.00/−1.00 × 10	0.15/0.00	Ø	2/0	400/480	Yes	Yes
A6	12/F	Anis	+0.25 DS+5.50/−1.50 × 40	0.00/0.30	Ø	2/2	800/800	Yes	Yes
A7	12/M	Anis	Plano+4.50 DS	0.00/0.15	Ø	2/1	400/800	Yes	Yes
A8	9/M	Anis	Plano−2.50/−1.75 × 160	−0.10/0.10	Ø	0/0	100/120	Yes	No
A9	9/M	Anis	+0.50/−1.50 × 180+2.50/−3.75 × 5	−0.10/0.10	Ø	0/1	200/240	Yes	Yes
A10	7/M	Anis	+2.25/−0.75 × 5+0.75/−0.50 × 180	0.15/0.00	Ø	0/0	30/60	Yes	Yes
A11	9/F	Anis	+0.50 DS+2.25/+0.50 × 100	0.00/0.30	Ø	2/2	800/800	Yes	Yes
A12	6/M	Anis	+0.50 DS+4.00/−1.25 × 180	0.00/0.15	Ø	2/1	800/800	Yes	Yes
A13	11/F	Mix	+7.00/−1.25 × 180+4.00 DS	0.15/0.00	ET15△Ø	1/0	200/240	Yes	Yes
A14	8/F	Mix	+1.00 DS+2.50 DS	0.00/0.15	ØET24△	2/0	400/480	Yes	Yes
A15	9/F	Mix	+1.50/−0.50 × 10+3.50/−0.75 × 180	−0.10/0.10	Ø XT15△	2/0	400/800	Yes	Yes
A16	5/F	Mix	+7.00/−1.25 × 175+5.50/−1.75 × 5	0.40/0.10	Ø^c^	2/2	800/800	Yes	Yes
A17	9/F	Mix	+1.50/−1.00 × 180−0.75 DC × 180	0.10/−0.10	Ø^c^	2/0	200/120	Yes	Yes
A18	11/M	Mix	+0.75/−0.50 × 45+5.75/−0.50 × 135	0.00/0.30	Ø ET30△	2/2	800/800	Yes	Yes
A19	11/M	Mix	−2.75/−2.00 × 180−0.75 DS	0.30/0.00	XT10△Ø	2/0	400/800	Yes	Yes
A20	12/M	Mix	Plano+2.00/−2.00 × 180	0.00/0.30	Ø ET35△	2/2	800/800	Yes	Yes
A21	6/M	Mix	+1.50/−0.75 × 180+4.50/−4.50 × 180	0.05/0.40	Ø^c^	2/0	800/800	Yes	Yes
A22	12/M	Stra	Plano+0.75/−1.25 × 180	0.00/0.30	Ø^c^	2/2	400/800	Yes	Yes
A23	5/F	Stra	−0.50 DS−0.50 DS	0.20/0.05	XT25△Ø	2/2	800/800	Yes	Yes
A24	5/F	Stra	+4.50/−0.50 × 180+5.50 DS	−0.10/0.10	Ø ET30△	0/0	400/480	Yes	Yes
A25	11/M	Stra	+1.50 DS+0.75 DS	0.50/0.05	Ø^c^	2/2	800/800	Yes	Yes
A26	7/M	Stra	+5.00 DS+6.00/−0.50 × 180	0.00/0.15	Ø ET35△	1/0	120/120	Yes	No
A27	8/F	Stra	+4.50/−0.75 × 170+4.75 DS	−0.10/0.10	ØET30△	0/0	400/480	Yes	No
A28	9/F	Stra	+7.50/−1.50 × 145+6.75/−0.75 × 40	0.15/0.00	ET15△Ø	0/0	120/240	Yes	No
A29	10/F	Stra	−1.00 DC × 170+0.75/−0.75 × 180	−0.10/0.10	Ø	0/0	60/240	Yes	No
A30	24/M	Anis	−14.25/−1.00 × 180−7.75/−0.75 × 10	0.70/0.00	Ø	1/0	400/800	Yes	No
A31	23/F	Anis	−6.75/−0.50 × 175−5.50/−1.00 × 170	0.20/−0.10	Ø	0/0	100/120	Yes	No
A32	20/F	Anis	−1.50/−4.50 × 180+1.00/−6.00 × 175	0.10/0.20	Ø	0/0	400/480	Yes	No
A33	9/M	Anis	+1.50/−0.50 × 30+6.00/−1.00 × 165	0.00/0.70	Ø	2/0	800/800	Yes	Yes
A34	8/M	Anis	+0.75/−0.75 × 175−1.50/−4.00 × 180	0.00/0.10	Ø	0/0	100/120	Yes	No

OD = right eye; OS = left eye; Anis = anisometropia; Stra = strabismus; Mix = mixed (strabismus, anisometropia); XT = exotropia; ET = esotropia.

a The classical Worth-4-Dot test was performed at far (5 m) and near (33 cm) distances. In this test, participants were asked to report the number and color of the dots that they saw under the photopic (35 cd/m^2^) viewing condition. Individuals’ interocular suppression indexes were assigned as: 0 (means no suppression) if four dots were reported and the perceived color of the bottom dot was white (some normal controls might report a rapid alternation of red and green of the bottom dot, they would also be classified as no suppression); 1 (means partial suppression) if four dots were reported and the perceived color of the bottom dot was either green or red and 2 (means complete suppression) if two or three dots were reported (Duke-Elder and Wybar, 1973).

b Stereo acuity of 800 arc secs was assigned for patients whose stereo acuity was too bad to be measured, that is, failed to pass the 480 arc secs level in the TNO test or the 400 arc secs level in the Optec3500 test.

c Strabismus had been corrected by surgery.

The normal controls had a normal or corrected to normal visual acuity in each eye (logMAR ≤0.00), normal stereo acuity (better than 60 seconds of arc) at near (tested with the TNO stereogram, 13th edition) and far (tested with the Optec 3500 inspection instrument) distances, absence of any ocular disease, strabismus, or binocular abnormalities. They had little to no spherical equivalent refractive errors (between −0.50 D and +0.50 D) or anisometropia (interocular spherical equivalent difference less than 1.00 D and cylindrical less than 0.50 D). We corrected all participants’ refractive errors during the test if they existed.

This study is in line with the Declaration of Helsinki and was approved by the review board of Wenzhou Medical University. Written consent forms were obtained from all participants’ parents or guardians before data collection.

### The Quantitative Interocular Suppression Test

**Figure 1.**
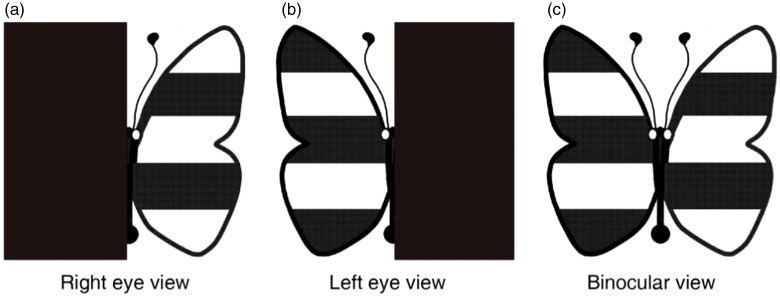
Visual stimuli for measuring interocular suppression. The butterfly’s left and right wings were dichoptically displayed with polarized glasses to the left and right eyes of the subjects, respectively: (a) The right wing of the target was only displayed to the right eye; (b) the left wing of the target was only displayed to the left eye; and (c) the fused perception from the stimuli seen by both eyes.

In the measurement, we used polarized glasses to dichoptically present a black–white colored (i.e., full contrast) butterfly stimulus (Figure 1) to observers. Observers were asked to report whether the left wing appeared as brightly as the right wing. If they did not appear equally bright, we asked the observers which of the two wings appeared brighter. We began the test without a ND filter (i.e., 0 ND). We then placed an ND filter selectively in front of the eye that perceived a *brighter* wing. The ND filter was adjusted from an optical density of 0.3 ND (transmittance of 50%) to 3 ND (transmittance of 0.098%), with a step size of 0.3 ND (in total, 10 levels; we provided a 2-minute adaptation for observers whenever the optical density was increased by 0.3 ND) until observers reported that the two wings were equally bright. The optical density (in ND) corresponding to that condition was used as a measure of the interocular suppression index. An interocular suppression index of 0 ND indicates a balanced suppression, while larger interocular suppression index values indicate more imbalance between eyes.

Observers’ interocular suppression was measured in a dimly lit room (15 lux) both at a far (5 m) and a near (33 cm) viewing distances. To minimize the effect of monocular acuity loss of the amblyopic eye, we made the size of the visual target relatively large: The visual target had a size of 1.81 cm × 1.81 cm (3.14° × 3.14°) at the near viewing distance (equivalent to the size of a Snellen acuity letter of 20/750) and 7.27 cm × 7.27 cm (0.8° × 0.8°) at the far viewing distance (equivalent to the size of a Snellen acuity letter of 20/200). It should be noted that the stimuli were printed on a clear Perspex sheet, with a back-light illumination of 320 cd/m^2^. As the size of the visual target was limited by the size of the Perspex sheet, we used a smaller angular size of the visual target at the far viewing distance than that at the near viewing distance. A chin rest was provided during the test to reduce head movements. Practice trials were provided before the test to ensure all the participants understood the test. We measured 20 of the amblyopes (A1–A20) and 12 of the normal controls twice in 1 to 2 weeks to assess the test–retest reliability. The same experimenter completed both the first- and retest. However, the experimenter was blind to the results of the previous test during the retest because we ensured that the records were kept by another experimenter.

### Worth-4-Dot Test

The classical Worth-4-Dot test was performed at far (5 m) and near (33 cm) distances. During the test, participants were asked to report the number and color of the dots that they saw under the photopic viewing condition (220 lux). The luminance of the Worth-4-Dot display was 35 cd/m^2^. Individuals’ interocular suppression indexes were assigned as: 0 (means no suppression) if four dots were reported and the perceived color of the bottom dot was white (some normal controls might report a rapid alternation of red and green of the bottom dot, they would also be classified as no suppression); 1 (means partial suppression) if four dots were reported and the perceived color of the bottom dot was either green or red and 2 (means complete suppression) if two or three dots were reported (Duke-Elder and Wybar, 1973).

### Stereo Acuity Test

Distance stereopsis was measured using the Optec3500 inspection instrument (Stereo optical Co. Inc., Chicago, IL). Near (33 cm) stereo acuity was measured using the TNO stereogram (TNO 13, Lameris Ootech BV, Celsiusbaan 6B, 3439 NC, Nieuwegein, the Netherlands).

### Binocular Phase Combination Test

Five of the patients (A30–A34) also participated in an additional test; we implemented this additional session to assess the relationship between the suppression index measured with our test (luminance-based) at the far distance of 5 m and that measured with a binocular phase combination task (contrast-based) at a simulated far viewing distance through 3D goggles. In the binocular phase combination task, two horizontal sign-wave gratings (0.46 cycles/degree) with equal and opposite phase-shift of 22.5° from horizontal of the screen were dichoptically presented to observers through 3D goggles. Observers were asked to adjust a reference line to report the binocular perceived phase at different interocular contrast ratios (i.e., 0, 0.1, 0.2, 0.4, 0.8, and 1) when the contrast of the grating input to the amblyopic eye was fixed at 100%. The measurement normally took about 30 minutes to finish. Observers’ interocular suppression indexes were quantified by the interocular contrast ratio when the two eyes were balanced in binocular phase combination (i.e., when the binocular perceived phase was 0°). An interocular suppression index of 1 indicates balanced eyes, while the smaller the interocular suppression index indicates the more imbalance between eyes.

### Statistical Analysis

We conducted an unpaired Wilcoxon rank sum test to compare the interocular suppression between amblyopes and controls. We performed a paired Wilcoxon rank sum test to compare near and far measurements within participant groups. We used a Spearman rank correlation analysis to assess the relationship between the interocular suppression measured with the butterfly test and the clinical measures, including the visual acuity of the amblyopic eye, interocular visual acuity difference, the stereo acuity, and the Worth-4-Dot test. We used a Bland-Altman plot and Spearman rank correlation to assess the test–retest reliability. We performed statistical analyses using the SPSS 19.0 software package (SPSS Inc., Chicago, IL, USA). *p* values < .05 were considered to be significant.

## Results

### More Interocular Suppression in Children With Amblyopia Than in Age-Matched Controls

**Figure 2.**
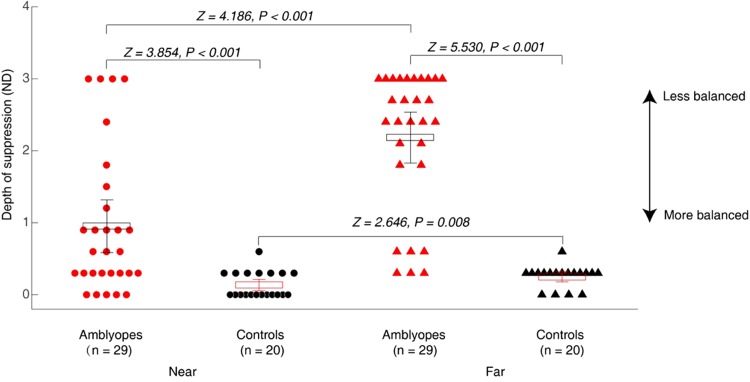
Interocular suppression in children with amblyopia and age-matched controls. Patients A1 to A29 and the age-matched normal controls (*n *=* *20) participated. Observers’ interocular suppression was measured at both a near (33 cm) and a far (5 m) viewing distances. Larger values in our suppression test (*y*-axis) indicate greater suppression. Red solid circle: amblyopes at near (*n *=* *29); black solid circle: controls at near (*n *=* *20); red solid triangle: amblyopes at far (*n *=* *29); black solid triangle: controls at far (*n *=* *20). Wilcoxon rank-sum tests were used to compare the interocular suppression between amblyopes and controls. Significantly more suppression was found in amblyopes at both the near and far viewing distances. The suppression at far viewing distance was significantly different from that at near viewing distance in both groups. Error bars indicate standard errors.ND = neutral density.

Figure 2 shows a plot of the depth of interocular suppression obtained from our luminance-based butterfly suppression test at near and far viewing distances for Patients A1 to A29 and the age-matched controls (*n *=* *20). As expected, there was little to no suppression in controls: 0.14 ± 0.18 (mean ± *SD*) at near; 0.24 ± 0.16 at far. Significantly more suppression was found in amblyopes than in controls at both the near (0.95 ± 1.00 vs. 0.14 ± 0.18; Z = 3.854, *p* < .001) and far (2.18 ± 0.97 vs. 0.24 ± 0.16; Z = 5.530, *p* < .001) viewing distances. The suppression at a far viewing distance was significantly different from that at a near viewing distance in both amblyopes (2.18 ± 0.97 vs. 0.95 ± 1.00; Z = 4.186, *p* < .001) and controls (0.24 ± 0.16 vs. 0.14 ± 0.18; Z = 2.646, *p* = .008).

### Good Test–Retest Reliability of Our Suppression Test

**Figure 3.**
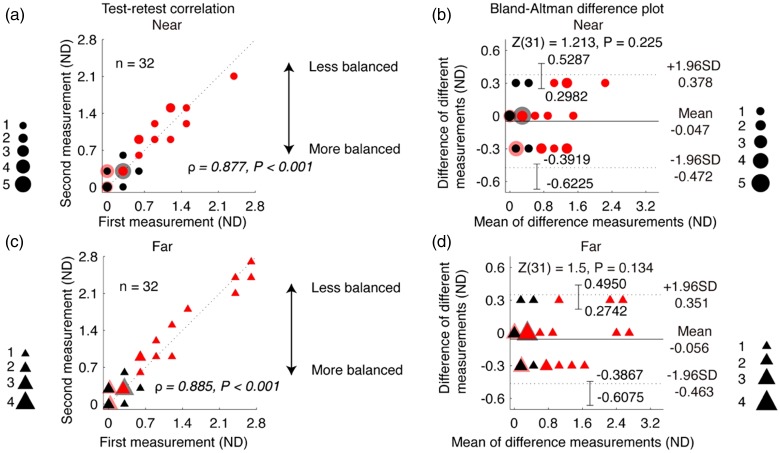
Test–retest reliability of our suppression test in children. Test–retest correlation (left column) and Bland-Altman difference plot (right column) for near and far distances. Twenty amblyopes (A1–A20) and 12 age-matched controls participated. Each dot represents results of one subject; the dashed line indicates the identity line. Results in the two tests were significantly correlated: for near (A), ρ = 0.877, *p* < .001; and for far (C), ρ = 0.885, *p* < .001. The mean difference between the two measures (i.e., the bias), indicated by the central black dashed line, was 0.047 for near (B) and 0.056 for far (D). Error bars in panels (B) and (D) represent 95% confidence limits for LOAs calculated using exact two-sided tolerance factor (Carkeet & Goh, 2018).ND = neutral density; *SD* = standard deviation.

We measured interocular suppression twice in 20 amblyopes (A1–A20) and 12 age-matched controls to assess the test–retest reliability of our suppression test. Figure 3 shows a plot of suppression measurements of the first test against those of the retest. We found a strong (for near, ρ = 0.877; for far, ρ = 0.855) and significant (for both near and far, *p* < .001) correlation (2-tailed Spearman’s correlation test) between the test and retest. We assess the reliability through a Bland-Altman plot, which shows little to no bias between the test and the retest: for near, bias = 0.047; for far, bias = 0.056, both were not significantly different from zero (for both cases, *p* > .74, 2-tailed Wilcoxon rank sum test). The 95% confidence limits of agreement (LoA), defined as 1.96 *SD* above and below the mean difference (Bland & Altman, 1999), were 0.378 and –0.472 ND in the near test and 0.351 and −0.463 ND in the far test, respectively. A Wilcoxon rank-sum test also showed that the difference between the two tests was not significant: for near, *Z *=* *1.213, *p* = .225; or far, *Z *=* *1.5, *p* = .134.

### Correlation Between the Interocular Suppression and the Clinical Characteristics of Amblyopes

**Figure 4.**
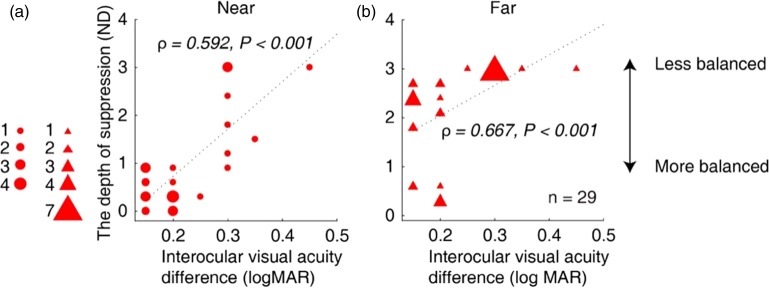
Relationship between the interocular suppression and the interocular visual acuity difference in children with amblyopia. Each dot represents results of one patient (Patients A1–A29 participated). We found significant correlations between the interocular suppression and interocular visual acuity difference: for near (A), ρ = 0.592, *p* < .001; and far (B), ρ = 0.667, *p* < .001.ND = neutral density.

**Figure 5.**
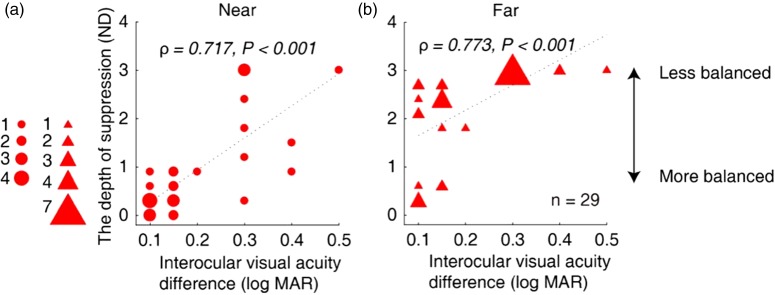
Relationship between the interocular suppression and the visual acuity of amblyopic eye in children with amblyopia. Each dot represents results of one patient (Patients A1–A29 participated). The statistical analysis for this comparison was conducted on the ranked data using Spearman’s rho. We found significant correlations between the interocular suppression and the visual acuity of amblyopic eye: for near (A), ρ = 0.717, *p* < .001; and for far (B), ρ = 0.773, *p* < .001.ND = neutral density.

Figure 4 shows a plot of the interocular visual acuity difference as a function of the interocular suppression that was measured at the near (Figure 4(a)) and the far (Figure 4(b)) viewing distances for Patients A1 to A29. We found significant correlations between the interocular suppression and the interocular visual acuity difference (for near, ρ = 0.592, *p* < .001; for far, ρ = 0.667, *p* < .001). We also found significant correlations between the interocular suppression and visual acuity of amblyopic eye (for near, ρ = 0.717, *p* < .001; for far, ρ = 0.773, *p* < .001; Figure 5). However, we did not find any significant correlation between the depth of suppression and degree of anisometropia (for near, ρ = 0.051, *p* = .793; for far, ρ = 0.248, *p* = .195).

**Figure 6.**
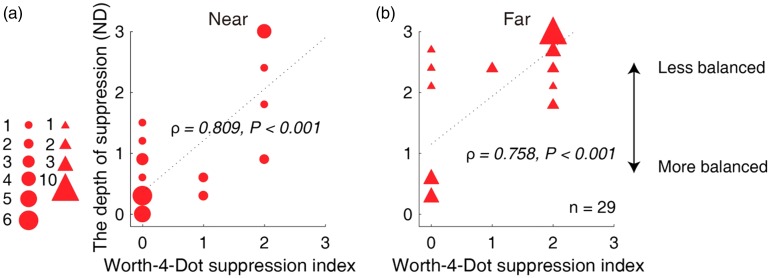
Relation between the measured depth of interocular suppression with our test and the Worth-4-Dot test in children with amblyopia. Larger values of our suppression tests (*y*-axis) are indicative of greater suppression. Lager values of the Worth-4-Dot tests (*x*-axis) are indicative of greater suppression. Each dot represents results from one patient (Patients A1–A29 participated). The statistical analysis for this comparison was conducted on the ranked data using Spearman’s rho. Positive correlation was found between interocular suppression measured with our quantitative detector and that with the Worth-4-Dot test: for near (A), ρ = 0.809, *p* < .001; for far (B), ρ = 0.758, *p* < .001.ND = neutral density.

We also conducted a correlation analysis between the interocular suppression measured with our test and that with the Worth-4-Dot test for Patients A1 to A29. As shown in Figure 6, there was a positive correlation between interocular suppression measured with our test and that with the Worth-4-Dot test: for near (A), ρ = 0.809, *p* < .001; for far (B), ρ = 0.758, *p* < .001.

**Figure 7.**
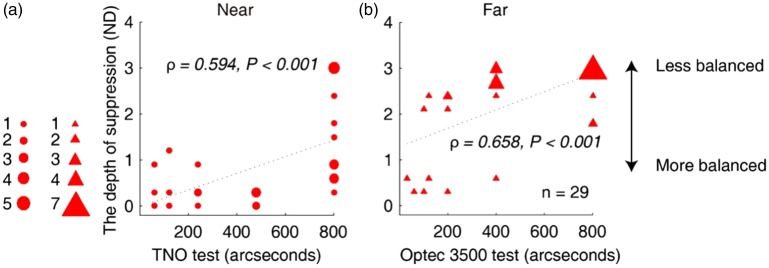
Relationship between the measured interocular suppression with our test and stereo acuity in children with amblyopia. Each dot represents results of one patient (Patients A1–A29 participated). The statistical analysis for this comparison was conducted on the ranked data using Spearman’s rho. Significant correlation was found between the measured interocular suppression using our quantitative detector and patients’ stereo acuity: TNO test (A), ρ = 0.594, *p* < .001; and Optec3500 test (B), ρ = 0.658, *p* < .001.ND = neutral density.

For Patients A1 to A29, there was also a significant correlation between the measured interocular suppression using our test and patients’ stereo acuity. As shown in Figure 7, patients who had greater interocular suppression had worse near (TNO test; ρ = 0.594, *p* < .001) and far (Optec3500 test; ρ = 0.658, *p* < .001) stereo acuity.

### Correlation Between the Interocular Suppression in Our Test With a Binocular Phase Combination Task

**Figure 8.**
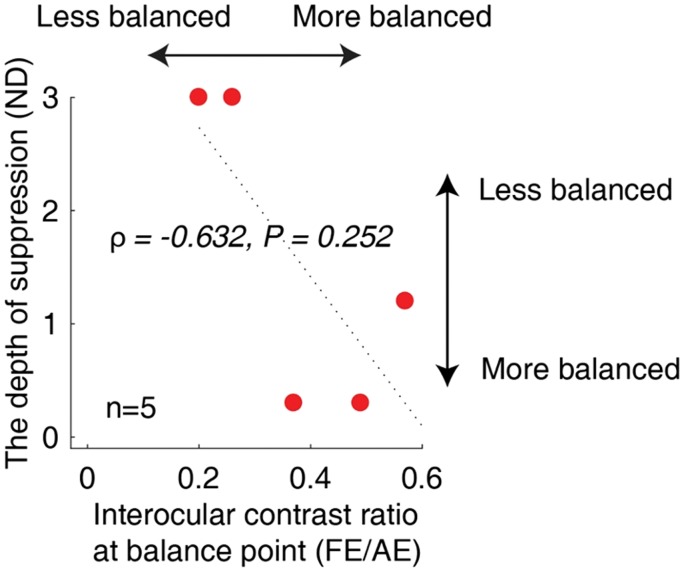
Relationship between the measured interocular suppression with our test and a binocular phase combination task. Five amblyopes (A30–A34) participated. Each dot represents results from one patient. There was a clear trend of negative correlation between the two tests, that is, patient who was less balanced in our test was also less balanced in the binocular phase combination. However, we did not find the correlation to be significant (ρ = −0.632, *p* = .252).ND = neutral density; FE = fellow-fixing eye; AE = amblyopic eye.

We were interested in whether there was a relationship between the suppression index that derived from our luminance-based interocular suppression test and that derived from the contrast-based laboratory tests existed. To answer this question, we recruited five amblyopes (A30–A34) and measured their interocular suppression using a binocular phase combination task. Figure 8 shows a plot of individuals’ interocular suppression index measured with our test as a function of that measured with the binocular phase combination task. There was a clear trend of negative correlation between the two tests (i.e., patient who was less balanced in our test was also less balanced in the binocular phase combination). However, we found that this correlation was not significant (ρ = −0.632, *p* = .252); this could be attributable to the small sample size.

## Discussion

Here, we introduced a child-friendly method to quantitatively measure the interocular suppression in amblyopic children. We had used findings that show that the degree of suppression could be modulated by the interocular luminance difference to design this method (Zhou, Jia, et al., 2013). The interocular suppression measured in a group of amblyopes was significantly correlated with other clinical measures, such as the visual acuity of amblyopic eye, interocular visual acuity difference, Worth-4-Dot test and stereo acuity.

Selectively reducing the luminance in one eye shifts the sensory eye dominance in favor of the eye with higher luminance in binocular viewings (Baker, Meese, Mansouri, & Hess, 2007; Chang et al., 2006; Gilchrist & Pardhan, 1987; Trick, Dawson, & Compton, 1982; Zhang, Bobier, Thompson, & Hess, 2011; Zhou & Hess, 2016; Zhou, Jia, et al., 2013). In this study, based on these previous findings, we quantified the interocular suppression in terms of the interocular luminance difference that were needed to balance the eyes in binocular viewing. We found that the interocular suppression significantly correlated with the severity of amblyopia (e.g., the visual acuity of the amblyopic eye, the interocular visual acuity difference). This finding is in line with those obtained from other laboratory techniques (Baker et al., 2008; Kwon et al., 2014; Li et al., 2010, 2011; Mansouri et al., 2008; Narasimhan et al., 2012; Zhou, Huang, et al., 2013). We also found that the interocular suppression significantly correlated with other clinical binocular tests, such as the Worth 4-Dot test, the TNO test, and the Optec 3500 test.

The different sizes of the stimuli at far and near, with fixed 2.5 cycles of black and white stripes, mean that the spatial frequency was higher in the far test than that in the near tests. Previous studies show that with increasing spatial frequency, observers’ two eyes become more imbalanced (Ding et al., 2013; Kwon et al., 2015; Y. Wang et al., 2019). The larger suppression at the far viewing distance may be accountable for this phenomenon. We compared the comparison between the suppression index that we measured with our test and with that measured with a laboratory test (i.e., the binocular phase combination) and found a clear trend of negative correlation between these two tests (i.e., patient whose two eyes were less balanced in our test were also less balanced in the binocular phase combination). However, such correlation was not significant (ρ = −0.632, *p* = .252), probably due to the small sample size. The inconsistent spatial frequencies between the two tests (3.12 cpd in the butterfly test vs. 0.46 cpd in the binocular phase combination task) might also have added some noise. We agree that the relationship between the contrast-based and luminance-based measures of suppression must be elucidated. Matched paradigms with matched stimuli should be developed in future.

In this study, we measured the interocular suppression at a dim light room (15 lux), Zhou and Hess (2016) showed that the effect of interocular luminance difference on the interocular suppression depends on the level of ambient light. Therefore, the interocular suppression may be different if the measurement takes place at another level of luminance. A setting with the same level of luminance is recommended if one wants to replicate/validate our experimental method.

During our test, 2 minutes were given for adaptation every time an ND filter was changed. In our study, we started from the 0 ND condition. It took about 2.5 to 25 minutes to finish the test, depending on the suppression of the patients (e.g., a 2.5-minute test for patients whose suppression index was 0.3 ND and a 25-minute test for patients whose suppression index was 3 ND). However, we recommend the use of a personalized starting point based on the severity of amblyopia to shorten the testing time. The advantage of our test is that it is convenient (could be completed in 2.5–25 minutes depending on the severity of the amblyopia) and suitable for testing children. Similar to the other child-friendly contrast-based tests (Birch et al., 2016; Mansouri et al., 2008; Narasimhan et al., 2012), it is a quantitative measure with a good test-retest reliability. The measured results highly correlated with the clinical tests that are currently in use and provided a much finer measure than that of Worth-4-dot (11 levels in our test vs. 3 levels in the Worth-4-dot). As finer measures of suppression could provide more information of the amblyopic binocular visual processing and enable better assessment of treatment (patching, training, etc.), we believe that it could be a good candidate for the clinical measurement of suppression in children with amblyopia.

## Conclusions

We conclude that our new test enables convenient and robust measurements of interocular suppression in children with amblyopia. The measured interocular suppression agrees with other clinical measures.
